# Changes in Areal Surface Textures Due to Ball Burnishing

**DOI:** 10.3390/ma16175904

**Published:** 2023-08-29

**Authors:** Slawomir Swirad

**Affiliations:** Faculty of Mechanical Engineering and Aeronautics, Rzeszow University of Technology, Powstancow Warszawy 8 Street, 35-959 Rzeszow, Poland; s.swirad@prz.edu.pl; Tel.: +48-17-865-1904

**Keywords:** ball burnishing, surface texture, burnishing pressure, burnishing width

## Abstract

The description of the areal texture of the surface is more comprehensive than that of roughness profiles. Ball burnishing led to an improvement in surface quality. In this work, the changes in areal surface textures due to ball burnishing were studied. Three surfaces of steel, two after milling and one after grinding, were subjected to ball burnishing. In the burnishing process, the burnishing pressure and width were variable parameters. Surface topographies before and after burnishing were measured using a white light interferometer. Ball burnishing was found to lead to a decrease in surface amplitude. The highest relative reduction was 94%. The changes in roughness height were greater with a higher amplitude of the surface texture before burnishing. The hybrid parameters also decreased as a result of ball burnishing. The characteristics of changes in spatial parameters mainly depended on the surface texture before burnishing.

## 1. Introduction

Surface topography substantially affects the functional properties of machine elements, such as contact between surfaces, sealing, friction, and wear [1,2,3]. The influence of surface texture on wear is important during running-in when accommodation between sliding surfaces occurs. However, this effect can also be maintained in a further period of machine part use. Previously, the behavior of the surface was analyzed on the basis of the parameters of roughness profiles [4]. However, the analysis of profiles can sometimes be insufficient [5]. Recently, an assessment of surface roughness was carried out based on the analysis of the results of areal (3D) surface topography [6,7]. Special parameters were developed for the analysis of the functional significance of the areal surface texture [8,9]. The analysis of areal surface texture is now very common in research, particularly after the development of efficient optical methods with a short measurement time [10]. Therefore, the researchers analyzed surface textures obtained after burnishing.

Skoczylas et al. [11] found that slide burnishing caused a reduction in the amplitude of the areal surface texture compared to the reference surface, after grinding. The obtained values of skewness Ssk and kurtosis Sku suggested that a good bearing surface was obtained. Ferencsik and Varga [12] studied the effect of parameters of diamond slide burnishing on the roughness height of the workpiece from the aluminum alloy. This analysis was restricted to roughness profile parameters. Kluz et al. [13] studied the effect of the parameters of slide burnishing (pressure, speed, and feed rate) on the Ra parameter of the steel shaft surface. Slide burnishing led to a reduction in surface roughness height. Korzynski and Zarski [14] found that slide diamond burnishing could reduce the Sa parameter of samples from AZ91 alloy after initial turning to 0.4 µm. Other amplitude parameters and hybrid parameters were also improved. Sachin et al. [15] studied the effects of slide diamond burnishing under a dry environment on the Ra parameter of the steel surface. Bataineh [16] obtained a reduction in roughness height by 87% of the sample from aluminum alloy by roller burnishing. Kurkute and Chavan [17] used a response methodology to minimize the Ra parameter after roller burnishing of aluminum alloy. Korzynski et al. [18] found that slide burnishing of valve stems led to decreases in surface height and slope and increases in peak density and correlation length.

Ball burnishing depends on the pressing of the hard ball to the workpiece during the sliding movement of the ball and during rolling friction contact. As a result of ball burnishing, plastic deformation of the treated surface occurred. The ball-burnishing process resulted in increases in hardness, the creation of compressive residual stresses, and surface smoothing. This technique can be an alternative to some finishing processes, such as grinding. Ball burnishing led to improvements in the tribological properties of machined elements [19,20,21,22,23]. Some functional properties caused by ball burnishing are related to surface texture. Therefore, researchers analyzed surface textures created by ball burnishing. Attabi et al. [19] reduced the surface amplitude by more than 90% via the application of ball burnishing. Banh et al. [24] simulated and experimentally verified the effect of ball burnishing on a decrease in the average height. The smallest roughness height was achieved for the medium value of the burnishing force. Dzionk et al. [25] applied burnishing by ceramic balls to hardened shafts. The relative reduction in the reduced peak height Spk was higher than that in the Sa parameter. Ball burnishing led to negative values of the Ssk parameter. Jerez-Mesa et al. [26] studied the effects of vibration-assisted ball burnishing of a sample from titanium alloy on the values of the parameters Sq, Ssk, and Sku. A reduction in roughness height was achieved due to burnishing. The values of skewness Ssk decreased towards zero. In some cases, surfaces with bimodal ordinate distribution were formed. Vaishya et al. [27] achieved a substantial decrease in the Sa parameter in the ball burnishing process due to an increase in the burnishing force. This effect was obtained for various speeds. Kanovic et al. [28] typically obtained a reduction in the Sa parameter due to the ball burnishing of steel samples. The smallest workpiece height was achieved when the depth of penetration was similar to the initial maximum surface height. A decrease in the burnishing feed and an increase in the ball diameter caused a reduction om the roughness height. In contrast, El-Tayeb et al. [20] obtained a decrease in the roughness height of the sample from aluminum alloy up to 75% by reducing the ball diameter. Swirad et al. [29] achieved a considerable reduction in the Sq parameter due to the burnishing of martensitic steel. Swirad et al. [21,30] found that the increase in the burnishing pressure of steel samples caused a decrease in the height of the roughness. However, for pressure that is too high, the amplitude of the surface texture increases. Cui et al. [31] studied the effect of pressure in the ball burnishing process of samples from Inconel 718 material on the change in roughness profile height. Kovavs et al. [32] analyzed the effects of various strategies of magnetic-assisted ball burnishing on parameters Ra, Rsk, and Rku of the steel surfaces. The cyclic strategy was found to create surfaces with the lowest Ra parameters in perpendicular directions. Capilla-Gonzales et al. [33] studied the effects of the ball-burnishing force and the number of tool passes on the average roughness Ra of a steel sheet surface. They obtained a reduction in roughness height of 82%.

The authors of previous articles [21,29,30] analyzed the effects of ball burnishing on various parameters of the areal (3D) surface texture.

Typically, during the analysis of the areal surface texture after ball burnishing, only height parameters were taken into account. In other cases, the impacts of only selected burnishing parameters on parameters of the areal surface texture were studied. A study of the wider set of parameters compared to the analysis of only height parameters is more comprehensive because not only height parameters are related to functional properties of machine elements. This work attempts to fill in the described niche. The fundamental aim of this work is to study changes in areal surface textures due to ball burnishing. The other aim is to analyze the effects of two burnishing parameters on the texture of burnished surfaces.

## 2. Materials and Methods

The experiments were performed on samples of X37CrMoV5-1 steel. This material may be used in machine parts operated at high temperatures, where burnished surfaces are applied, such as in injection molds. Table 1 presents the chemical composition of this material [34]. The machined specimens had a rectangular shape of 70 × 60 × 10 mm. They were milled and ground. Three samples were subjected to each treatment. The milled samples were machined using an insert face-milling cutter of 80 mm diameter. Inserts Groth SNMX1206 (Abplanalp, Warsaw, Poland) were used in this experiment. Two types of milled samples, M1 and M2, were obtained. Table 2 presents the machining parameters of them.

The samples after grinding G1 were machined using the aloxite disc type 99A60L5VBE (Andre Abrasive Articles, Kolo, Poland) at 3250 rpm, and a medium-quality surface roughness texture was achieved.

**Figure 1:**
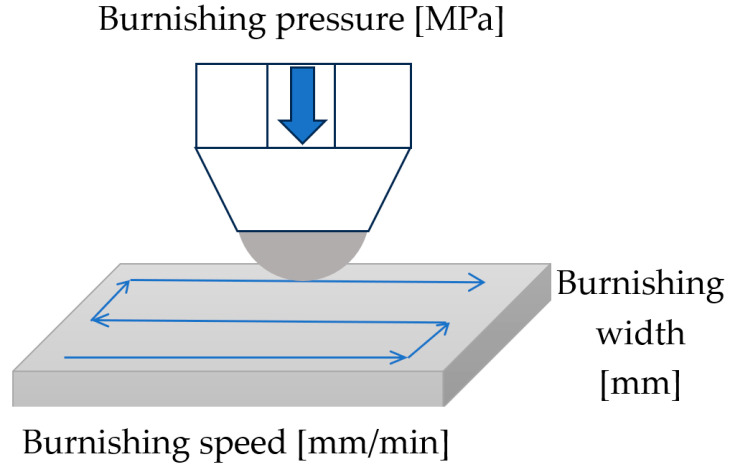
The scheme of the raster burnishing strategy process.

The samples after milling M1 and M2 and after grinding G1 were burnished using the Haas CNC Vertical Mill Center VF-1. Ball burnishing tests were conducted using the Ecoroll (Ecoroll AG Werkzeugtechnik, Celle, Germany) burnishing system. This system consists of a high-pressure pump and a burnishing tool 6 mm in diameter, which are connected via pressure hoses. The pressure was provided by a hydraulic pump. When the tool is fed along with the workpiece, the ball is pressed against the workpiece, resulting in the burnishing operation, and rolling friction contact occurs. The force of burnishing can be controlled by varying the hydraulic pressure of the fluid. Table 3 lists the burnishing parameters. They were selected on the basis of previous investigations of the author of this paper. The burnishing speed was constant because its effect on surface texture is small. Burnishing pressure p and width w (Figure 1) were input variables. They covered all the possible ranges of parameters possible to obtain using burnishing equipment. 

Areal (3D) surface topographies of machined samples before and after ball burnishing were measured using a white light interferometer Talysurf CCI Lite. Surfaces were only leveled without using digital filtration. The measuring area of 3.29 mm × 3.29 mm contained 1024 × 1024 data points.

Roughness profiles were also measured using a Talysurf i series stylus profilometer equipped with a tip radius of 2 µm. Each profile was measured at a speed of 0.5 mm/s.

## 3. Results and Discussion

**Figure 2:**
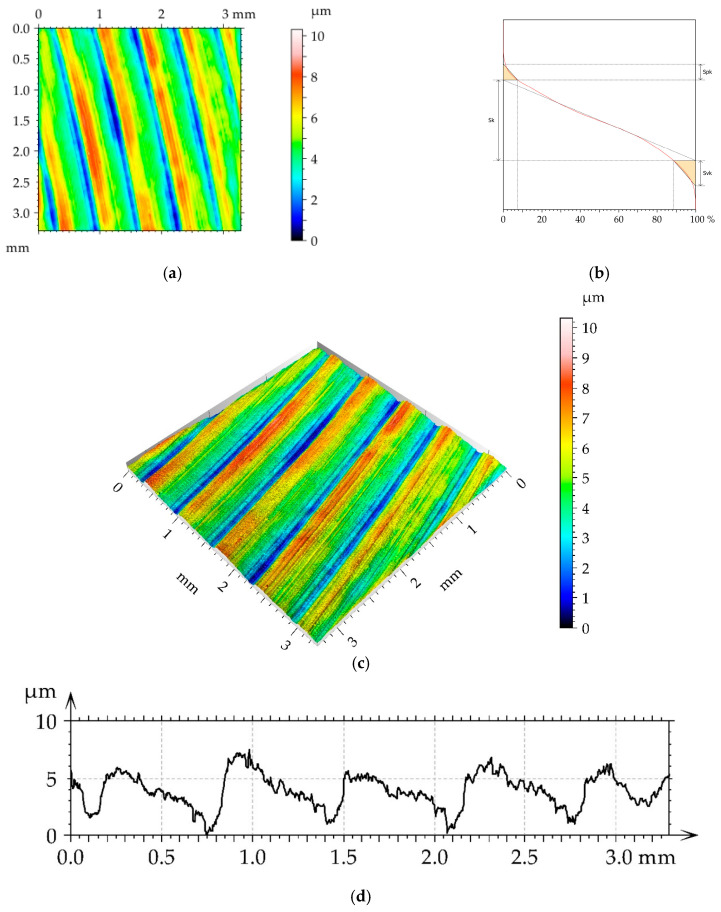
Pseudo-color image (**a**), material ratio curve (**b**), 3D view (**c**), and representative profile (**d**) of surface texture after milling M1.

Figure 2 presents the contour plot, isometric view, material ratio curve, and selected profile of the example of the surface texture of the M1milled sample. This surface had a deterministic character with an amount of random noise. The main wavelength was approximately 1.2 µm. Table 4 lists mean values and standard deviations σ of the surface texture parameters of the surfaces after milling M1 and the surfaces subjected to the burnishing process. The texture parameters were calculated according to the ISO 25178-2 standard [35]. The following parameters were analyzed: rms. height Sq, skewness Ssk, kurtosis Sku, maximum peak height Sp, maximum valley depth Sv, maximum height Sz, arithmetical mean height Sa, correlation length Sal, texture aspect ratio Str, rms. slope Sdq, developed interfacial areal ratio Sdr, peak density Spd, mean peak curvature Spc, core height Sk, reduced peak height Spk, and reduced valley depth Svk. The selected parameters describe various properties of the surface texture: Height, hybrid, and spatial. They are functionally important [9]. Height parameters are related to friction and wear. Contact between rough surfaces and lubrication depends on spatial surface properties. Hybrid parameters are related to friction, wear, rough surface contact, and the possibility of the creation of adhesive joints. Feature parameters are important in problems of contact between rough surfaces. The functional parameters are related to friction wear and resistance to seizure. Some parameters in this set describe one-process machined surfaces [36]. 

**Figure 3:**
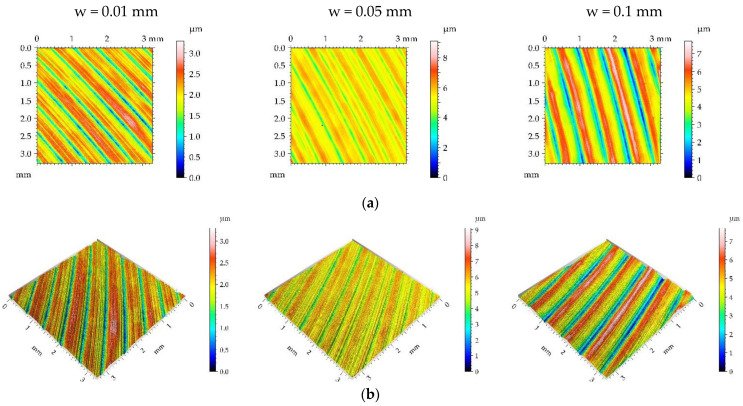

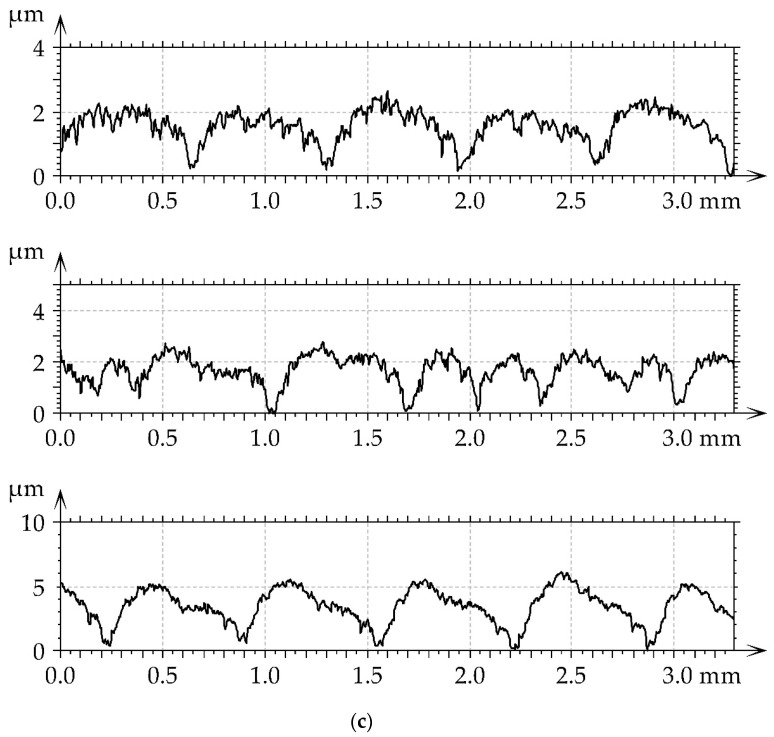
Pseudo-color images (**a**), 3D views (**b**), and profiles (**c**) of the M1 surface after burnishing surfaces with pressure p of 5 MPa and burnishing width w of 0.01 mm, 0.05, and 0.1 mm, respectively.

**Figure 4:**
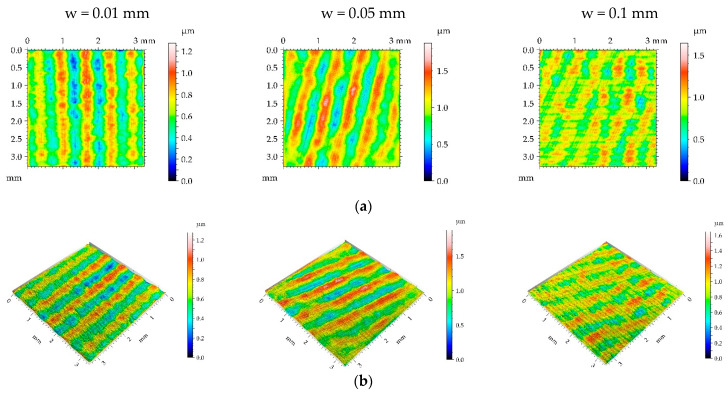

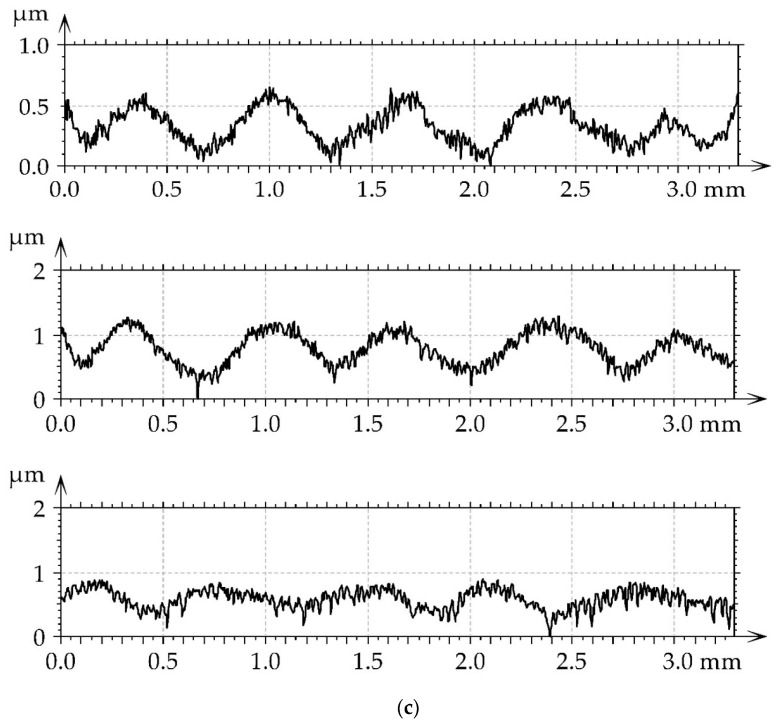
Pseudo-colour images (**a**), 3D views (**b**), and profiles (**c**) of the M1 surface after burnishing surfaces with pressure p of 15 MPa and burnishing width w of 0.01 mm, 0.05 mm, and 0.1 mm, respectively.

**Figure 5:**
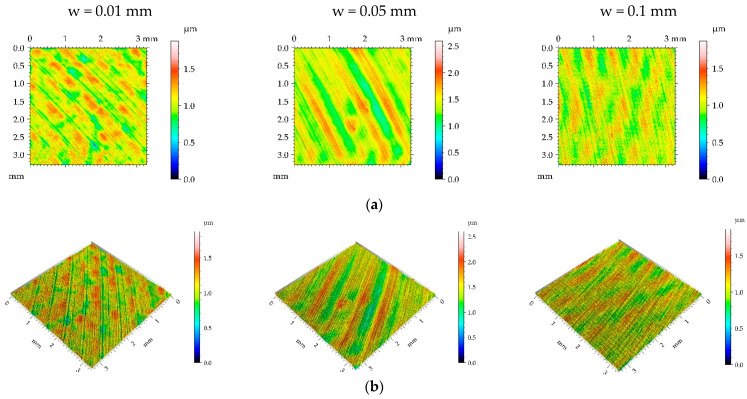

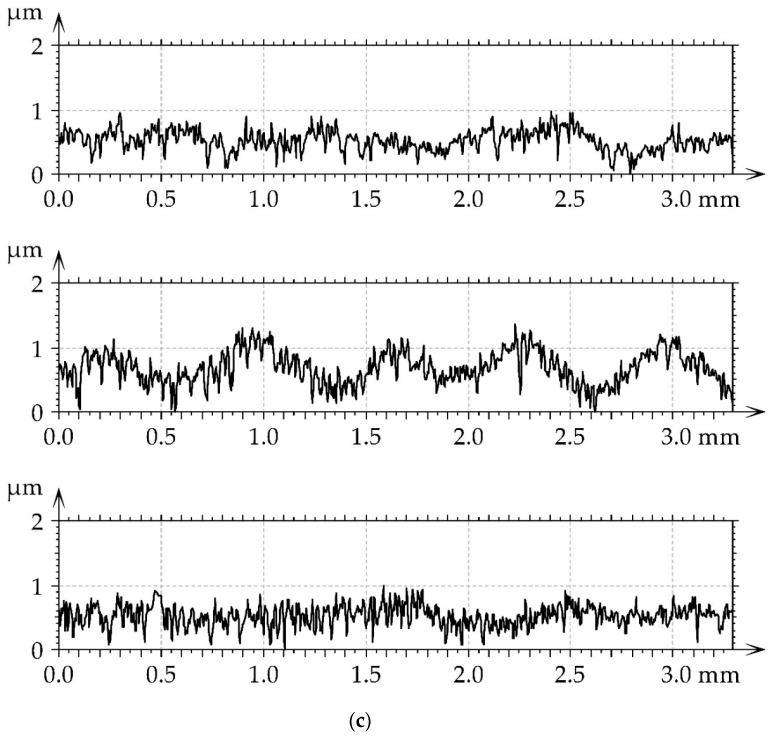
Pseudo-colour images (**a**), 3D views (**b**), and profiles (**c**) of the M1 surface after burnishing surfaces with pressure p of 25 MPa and burnishing width w of 0.01 mm, 0.05 mm, and 0.1 mm, respectively.

**Figure 6:**
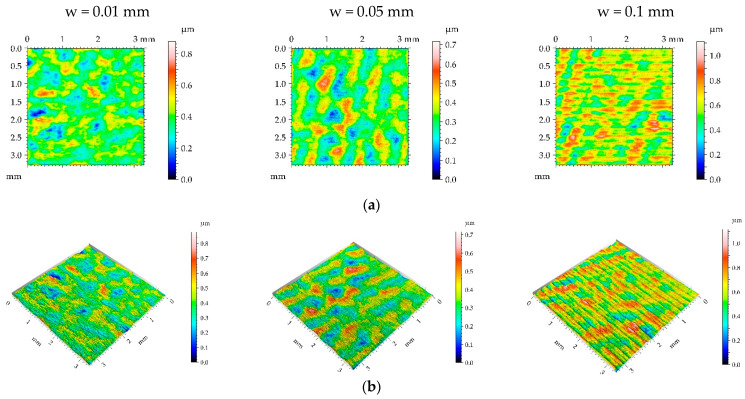

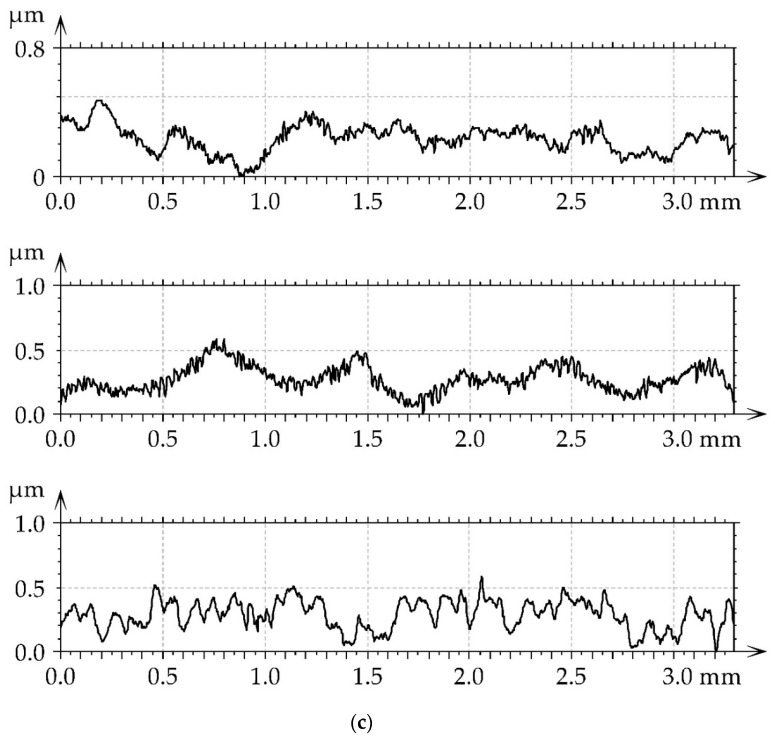
Pseudo-colour images (**a**), 3D views (**b**), and profiles (**c**) of the M1 surface after burnishing surfaces with pressure p of 35 MPa and burnishing width w of 0.01 mm, 0.05 mm, and 0.1 mm, respectively.

Figure 3, Figure 4, Figure 5 and Figure 6 present contour plots, isometric views, and selected profiles of the example of milled sample M1 subjected to ball burnishing. The Sku parameter of less than 3 of the milled sample M1 confirmed that this texture had a deterministic character. This texture is anisotropic, and the Str parameter is close to zero. One can see from the analysis of Figure 3 that burnished surfaces with widths of 0.01 and 0.05 mm had a random-deterministic character; however, a burnishing width of 0.1 mm led to a surface very similar to milled surface M1 (deterministic character with a small amount of a random component). The analysis of Table 4 confirmed this observation. Most of the parameters corresponding to the burnishing width of 0.1 mm are similar to those of the milled sample M1, and only the surface amplitude slightly decreased due to burnishing. The amplitude parameters of the surface texture burnished with smaller widths decreased, and this reduction was the highest for the smallest burnishing width. For example, the maximum decrease in the Sq parameter due to burnishing was 70%. The correlation lengths Sal and the texture aspect ratios Str of these samples decreased. Hybrid parameters and the mean peak curvature Spc decreased due to the reduction in surface roughness height. Peak density Spd increased for the smallest burnishing width and decreased for the medium burnishing width. The values of kurtosis Sku higher than 3 are typical for random textures. The Ssk skewness decreased, and the negative values of this parameter can be beneficial for the tribological performance of the machine elements [9].

One can see from the analysis of Figure 4 that most burnished surfaces with a pressure p of 15 MPa had a deterministic character, which was confirmed from the analysis of Table 4—the values of kurtosis were less than 3. Surface height decreased due to ball burnishing; the largest reduction occurred for the smallest burnishing width, while the smallest occurred for the medium width. The increase in burnishing pressure from 5 to 15 MPa caused a greater reduction in surface amplitude; maximum decreases in the Sq parameter were near 90%, compared to the milled texture. Unlike the smallest burnishing pressure, the spatial parameters increased due to burnishing. Due to a high roughness decrease and correlation length increase, a large reduction in the parameters Sdq, Sdr, and Spc occurred—the Sdr parameter decreased up to 96% due to ball burnishing. Because of the presence of noise (random component), the peak density Spd highly increased.

The surfaces burnished with a pressure p of 25 MPa have a random or random-deterministic character, as was evident from the analysis of Figure 5 and Table 4. Ball burnishing with a pressure of 25 MPa led to a similar roughness height to burnishing with a pressure of 15 MPa. The smallest reduction in the roughness height occurred for the medium burnishing pressure. There was not a clear tendency for changes in spatial parameters. Due to the existence of noise, the parameters Sdq and Spc were higher after burnishing with a pressure of 25 MPa compared to those after burnishing with a pressure of 15 MPa. Due to the presence of a random component, the peak density increased compared to the milled surface M1. The skewness values of the burnished surfaces were negative.

When the highest burnishing pressure p of 35 MPa was applied, the surfaces had a random or random-deterministic character (see Figure 6 and Table 4), and for the highest burnishing width, the profile appeared random. The reduction in the roughness height was the highest, and it was the lowest for the largest burnishing width. The highest decrease in the Sq parameter was greater than 99%. The spatial parameters increased, and the large growth in the Str parameter in a range of 0.41–0.53 was interesting. The burnished surface became mixed surfaces, not anisotropic. Similar to the roughness height, the reduction in the parameters Sdq, Sdr, and Spc was also high. For example, the relative decrease in the Sdr parameter may be greater than 99%. Similar to other analyzed surfaces, the peak density increased as a result of burnishing compared to that of the milled sample.

Generally, an increase in the burnishing pressure caused a greater reduction in the roughness height and hybrid parameters compared to the milled surface. The surface burnished with the highest pressure is characterized by the largest values of spatial parameters. The surface after milling M1 changed character from deterministic to random-deterministic or random when the burnishing pressure increased.

**Figure 7:**
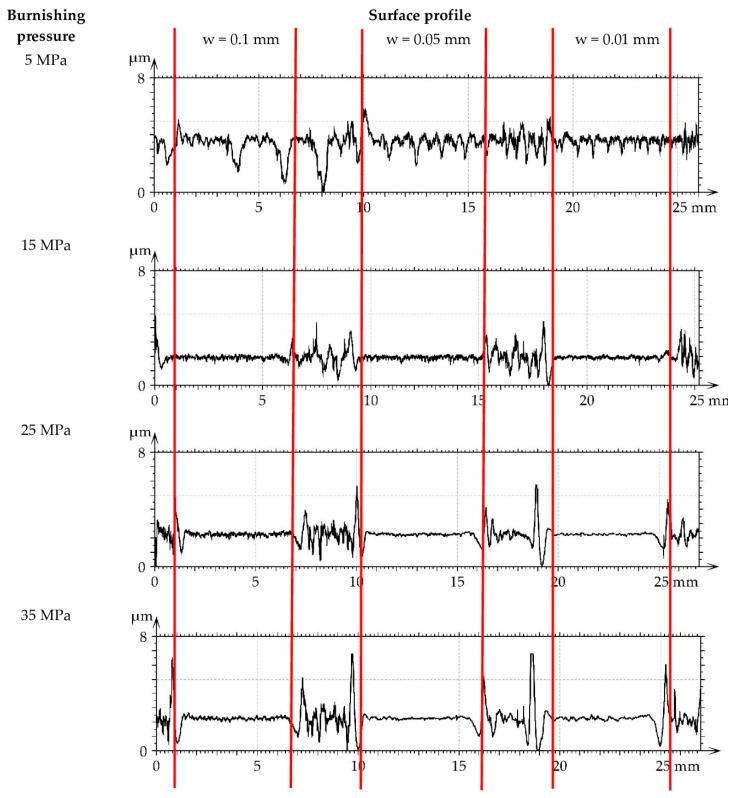
Roughness profiles performed through milled M1 and burnished samples.

Figure 7 presents roughness profiles performed through milled M1 and burnished samples. They were shown for direct comparison of surfaces after various treatments. A decrease in the roughness height of burnished surfaces can be observed with an increase in the burnishing pressure.

**Figure 8:**
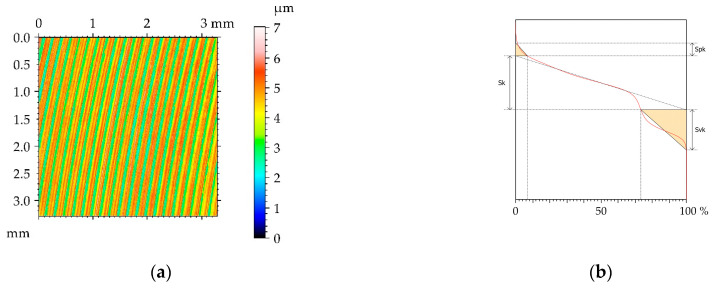

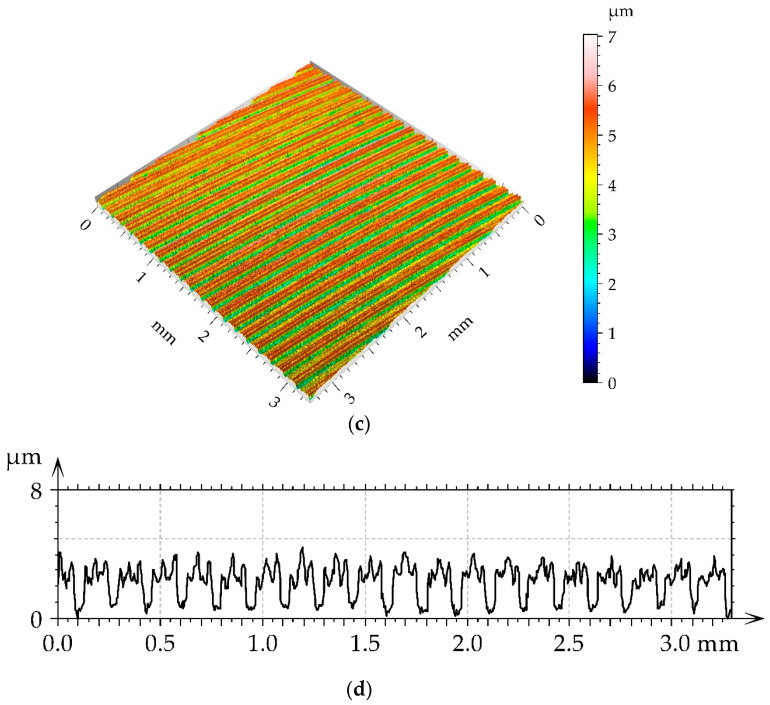
Pseudo-color image (**a**), material ratio curve (**b**), 3D view (**c**), and representative profile (**d**) of surface texture after milling M2.

Figure 8 presents pseudo-color image, isometric view, material ratio curve, and selected profile of the example of the surface texture of the milled sample M2. This anisotropic surface had a deterministic character. The amount of random components was marginal, which is evident after analysis of the material ratio curve. The main wavelength is approximately 0.2 µm, much smaller than that of the surface M1. The skewness Ssk is negative. The roughness height of surface M2 is also smaller than that of surface M1.

**Figure 9:**
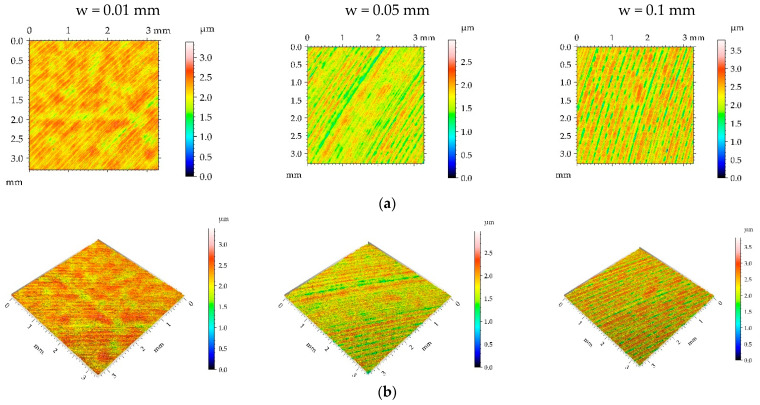

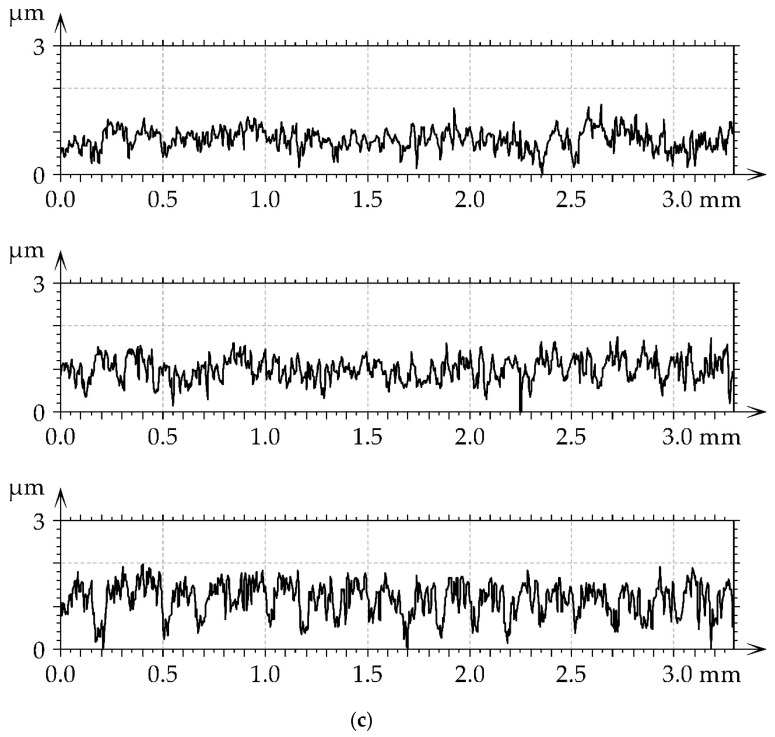
Pseudo-color images (**a**), 3D views (**b**), and profiles (**c**) of the M2 surface after burnishing surfaces with pressure p of 5 MPa and burnishing width w of 0.01 mm, 0.05 mm, and 0.1 mm, respectively.

**Figure 10:**
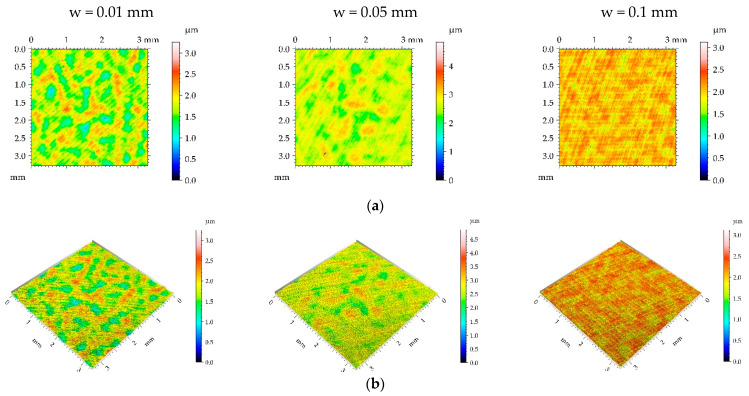

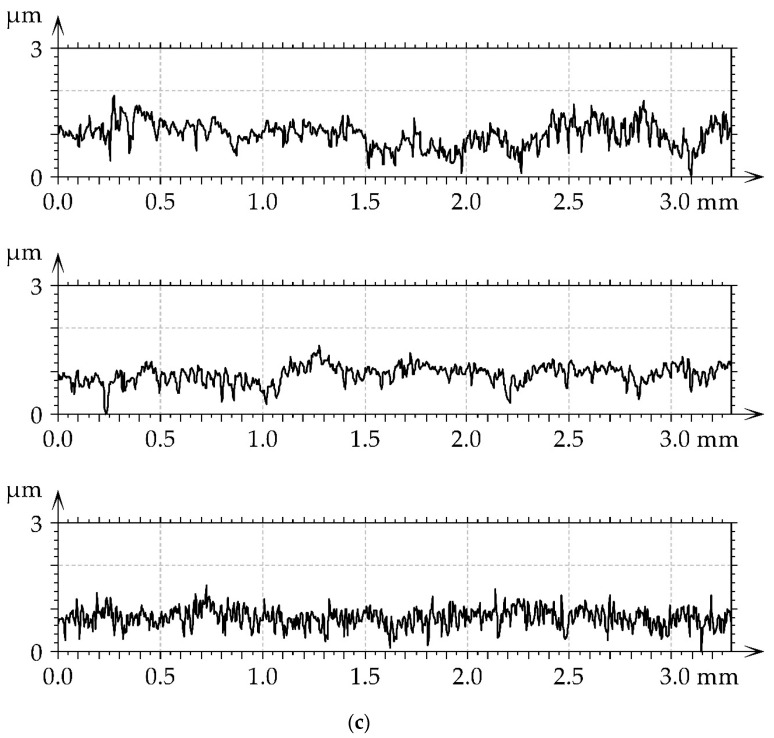
Pseudo-color images (**a**), 3D views (**b**), and profiles (**c**) of the M2 surface after burnishing surfaces with pressure p of 15 MPa and burnishing width w of 0.01 mm, 0.05 mm, and 0.1 mm, respectively.

**Figure 11:**
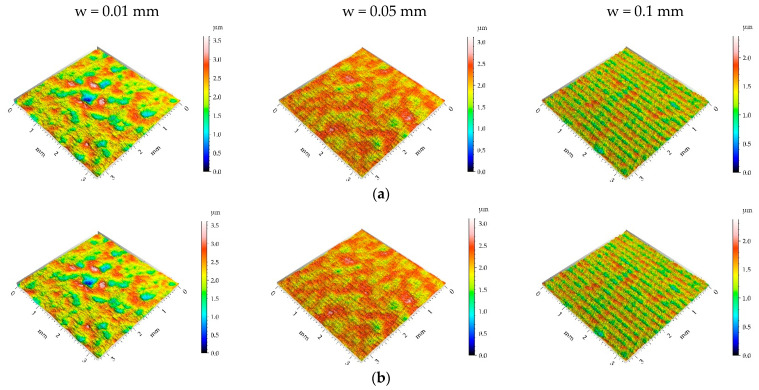

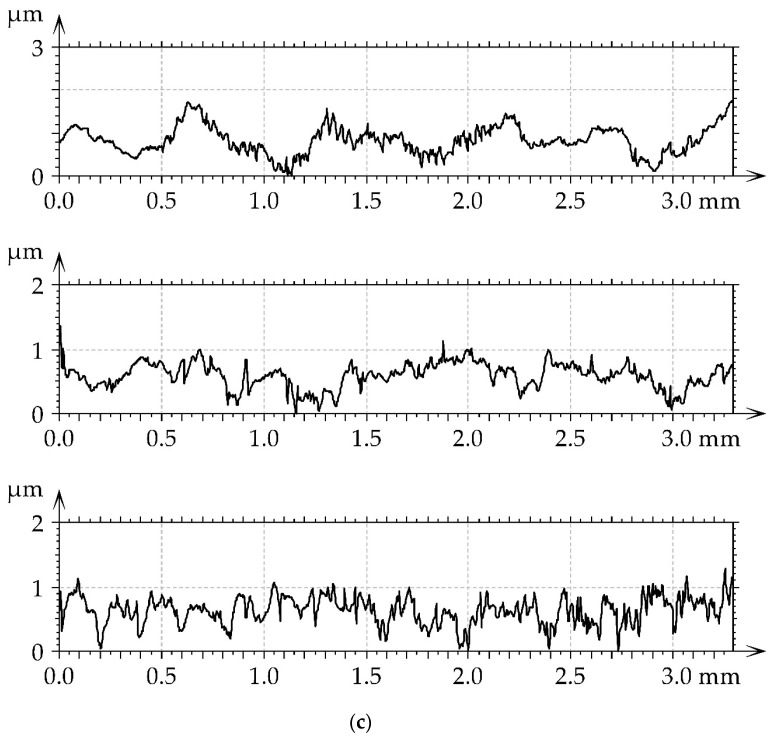
Pseudo-color images (**a**), 3D views (**b**), and profiles (**c**) of the M2 surface after burnishing surfaces with pressure p of 25 MPa and burnishing width w of 0.01 mm, 0.05 mm, and 0.1 mm, respectively.

**Figure 12:**
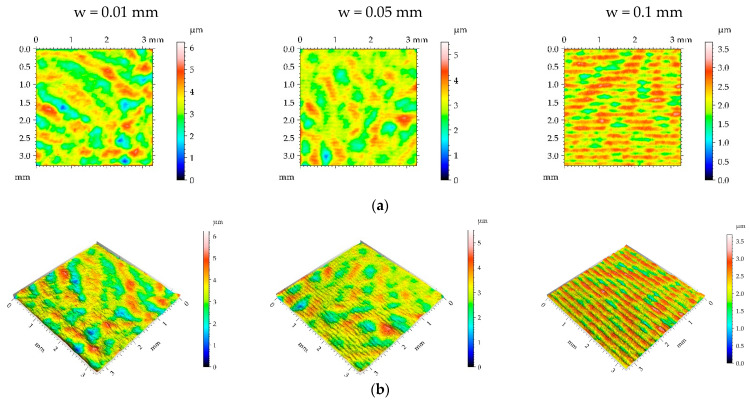

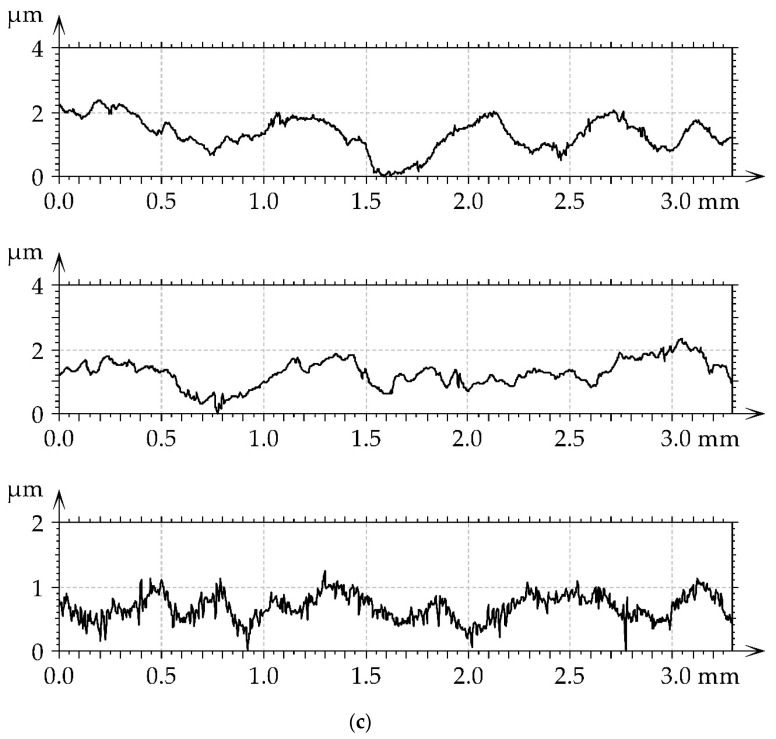
Pseudo-color images (**a**), 3D views (**b**), and profiles (**c**) of the M2 surface after burnishing surfaces with pressure p of 35 MPa and burnishing width w of 0.01 mm, 0.05 mm, and 0.1 mm, respectively.

Table 5 lists the mean values and standard deviations σ of the surface texture parameters of the surfaces after milling M2 and the surfaces subjected to the burnishing process. Figure 9, Figure 10, Figure 11 and Figure 12 present pseudo-color images, 3D views, and selected profiles of representative examples of the milled M2 sample subjected to ball burnishing. 

The application of the smallest burnishing pressure p caused a decrease in roughness height (Figure 9 and Table 5). The smallest height reduction occurred for the highest burnishing width. The largest decrease in the Sq parameter due to the application of ball burnishing was 77%. Although the surface height was considerably reduced compared to the milled surface, the spatial characteristic remained unchanged. Changes in Sal and Str parameters due to burnishing were small. Due to the burnishing, the character of the surface changed from deterministic to random. Only for the largest burnishing width was the deterministic part visible in Figure 9. As a result of the reduction in surface height, the hybrid parameters were also reduced. However, the decrease in the Spc parameter was smaller than that of hybrid parameters. Due to the change in the surface character from periodic to random, the peak density of the burnished surfaces increased, compared to the milled sample.

When the burnishing pressure p of 15 MPa was applied, the spatial surface characteristic was rather unchanged for the largest burnishing width (Figure 10 and Table 5). In other cases, surfaces changed towards those typically achieved during the burnishing of random or random-deterministic characters—Sal and particularly Str increased, and the surfaces changed from anisotropic to random. The surface amplitude was similar to that obtained using a pressure of 5 MPa; the smallest roughness height was obtained for the largest burnishing width. Due to burnishing, the hybrid parameters and Spc decreased and peak density Spd increased similarly to surfaces produced using the burnishing pressure of 5 MPa. 

For the burnishing pressure p of 25 MPa, similar to the surface obtained with a burnishing pressure of 15 MPa, the spatial characteristic of the surface obtained for the highest burnishing width was similar to the milled M2 surface (Figure 11 and Table 5), and the changes in the Sal and Str parameters were small. In contrast, the smallest burnishing width led to different texture characteristics, an aroused isotropic surface (Str = 0.9), and a lower peak density compared to the milled surface due to the small amount of the random component. For a medium burnishing width, the spatial parameters also increased compared to the milled surface. The surface height was rather similar to those obtained for the lower burnishing pressure; only the smallest burnishing width led to a higher roughness amplitude. The hybrid parameters considerably decreased; the highest decrease in the Sdr parameter was obtained for the smallest burnishing width of 97%, which was caused by a decrease in amplitude and an increase in the correlation length. Peak density increased for medium and large burnishing widths.

When the highest burnishing pressure was applied, mixed and isotropic surfaces were obtained (Str = 0.19–0.77). The highest amount of random components (Figure 12 and Table 5) of a smaller wavelength was obtained for the highest burnishing width, which resulted in the smallest correlation length of Sal and the highest peak density of Spd. For other burnishing widths, the correlation length Sal increased more and the peak density decreased compared to the milled surface M2. The height of the roughness was greater compared to other burnishing pressures; the highest decrease in the Sq parameter of 65% was obtained for the highest burnishing width. The smallest burnishing width led to the highest roughness. Due to the reduction in amplitude and increase in correlation length, the hybrid parameters considerably decreased for burnishing widths of 0.01 and 0.05 mm, and the reduction in the Spc parameter was smaller.

The application of burnishing pressures of 5, 15, and 25 MPa led to similar roughness heights. A further increase in pressure caused an increase in the amplitude of roughness. The spatial parameters increased due to ball burnishing. The highest reduction in roughness and the lowest change in spatial characteristics occurred for the highest burnishing width. Surface textures with negative skewness were achieved, and the highest values were obtained for the highest burnishing pressure. 

**Figure 13:**
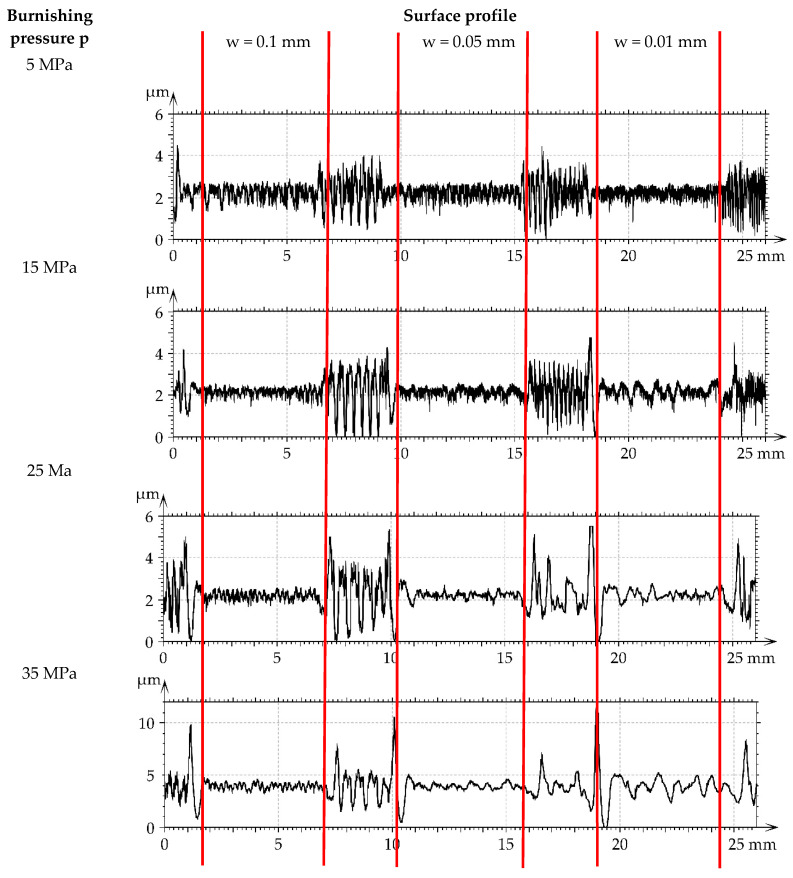
Roughness profiles performed through milled M2 and burnished samples.

Figure 13 presents roughness profiles performed through milled M1 and burnished samples. A large number of short-wavelength components can be observed in profile details corresponding to the highest burnishing pressure.

**Figure 14:**
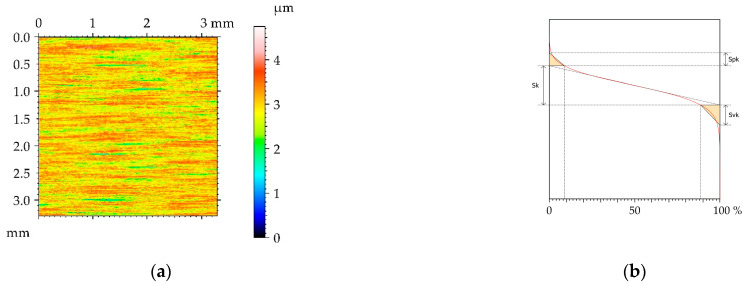

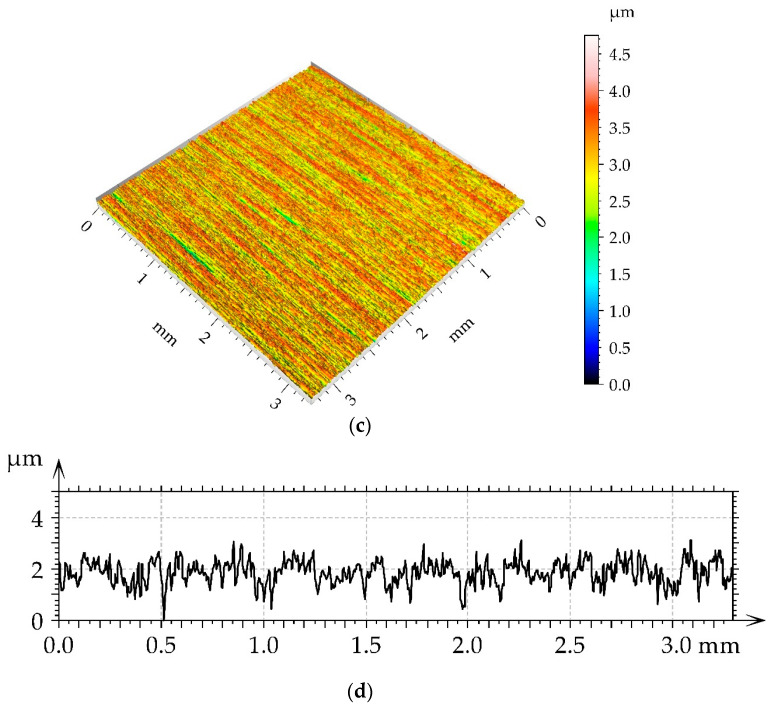
Pseudo-color image **(a**), material ratio curve (**b**), 3D view (**c**), and representative profile (**d**) of surface texture after milling G1.

Figure 14 presents the contour plot, isometric view, material ratio curve, and selected profile of the example of the surface texture of the ground sample G1. This anisotropic surface had a random character, which is evident after analysis of the material ratio curve. The skewness Ssk is negative, which is a characteristic feature of the surface after grinding [3]. The roughness height, determined by the Sq parameter, is 0.417 µm. 

**Figure 15:**
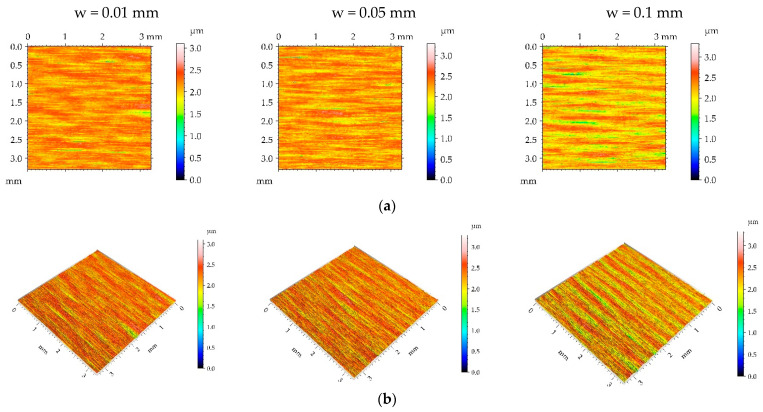

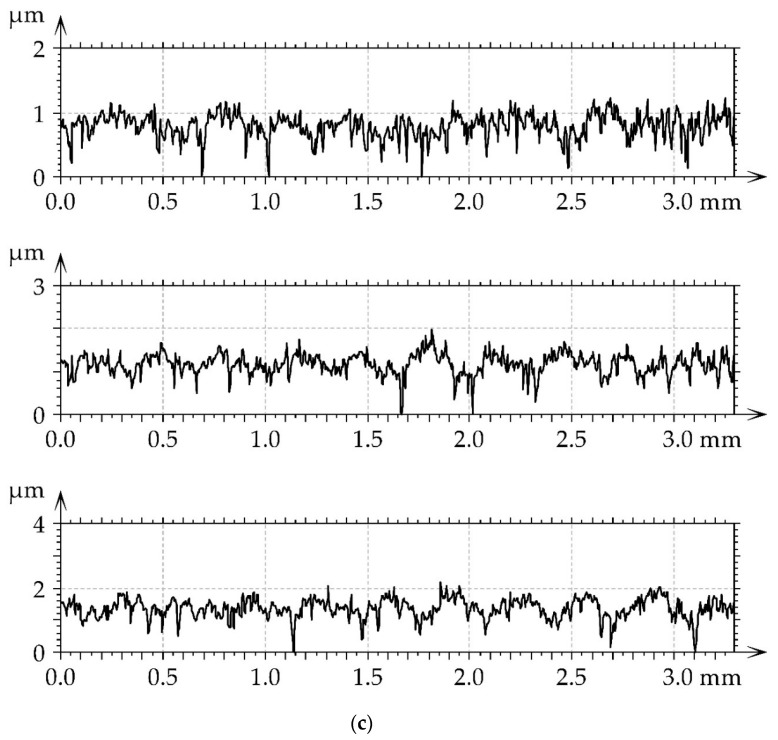
Pseudo-color images (**a**), 3D views (**b**), and profiles (**c**) of the G1 surface after burnishing surfaces with pressure p of 5 MPa and burnishing width w of 0.01 mm, 0.05 mm, and 0.1 mm, respectively.

**Figure 16:**
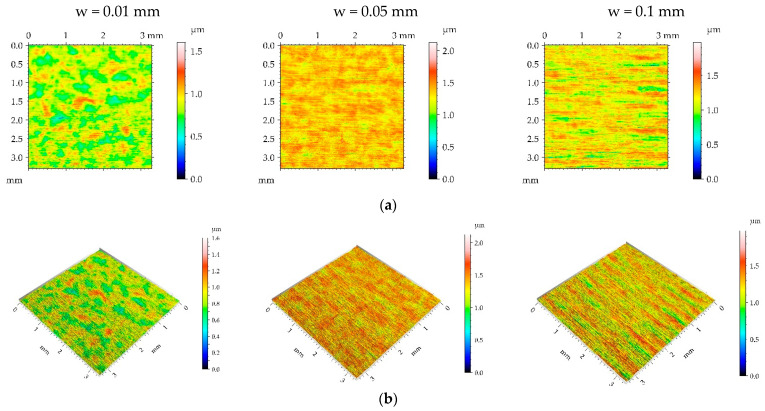

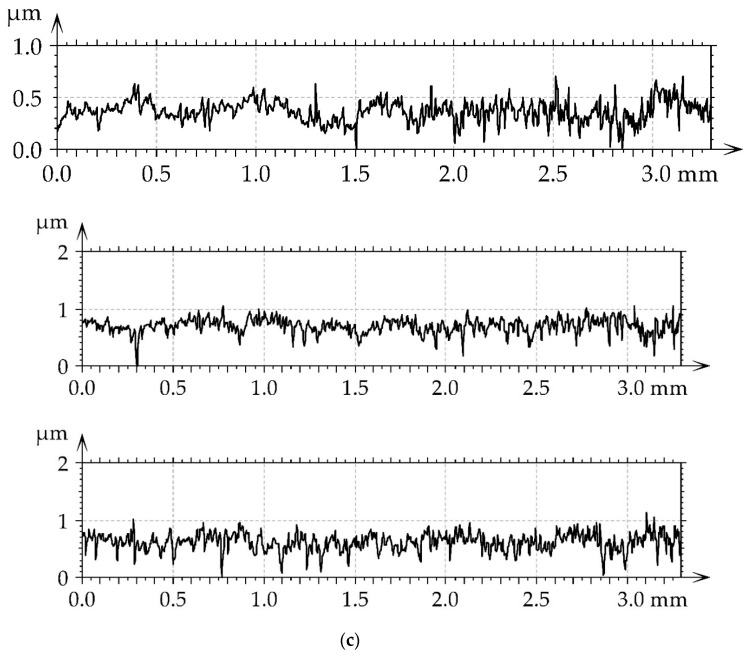
Pseudo-color images (**a**), 3D views (**b**), and profiles (**c**) of the G1 surface after burnishing surfaces with pressure p of 15 MPa and burnishing width w of 0.01 mm, 0.05 mm, and 0.1 mm, respectively.

**Figure 17:**
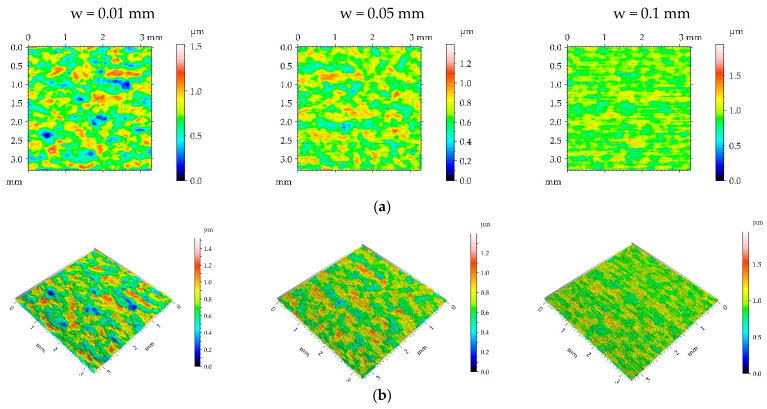

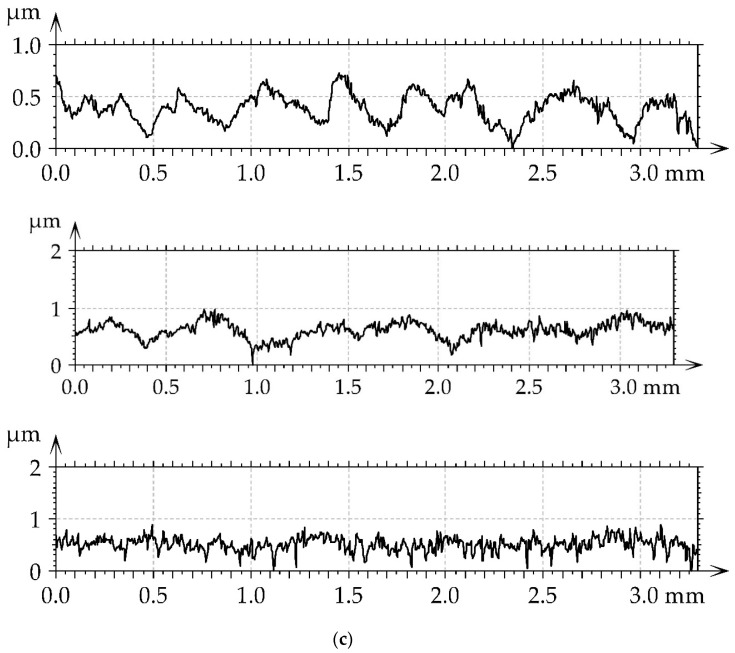
Pseudo-colour images (**a**), 3D views (**b**), and profiles (**c**) of the G1 surface after burnishing surfaces with pressure p of 25 MPa and burnishing width w of 0.01 mm, 0.05 mm, and 0.1 mm, respectively.

**Figure 18:**
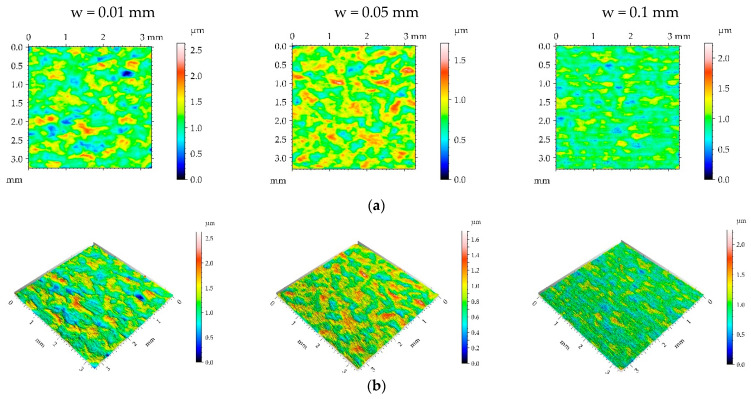

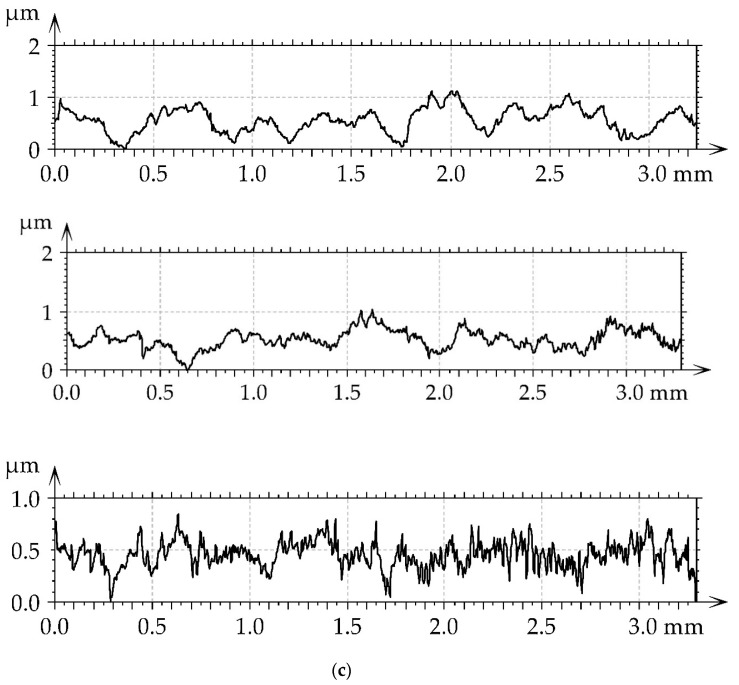
Pseudo-color images (**a**), 3D views (**b**), and profiles (**c**) of the G1 surface after burnishing surfaces with pressure p of 35 MPa and burnishing width w of 0.01 mm, 0.05 mm, and 0.1 mm, respectively.

Table 6 lists the mean values and standard deviations σ of the surface texture parameter of the surfaces after grinding G1 and the surfaces subjected to the burnishing process. Figure 15, Figure 16, Figure 17 and Figure 18 present the contour plot, isometric view, and selected profile of the example of the G1 sample subjected to ball burnishing. 

When the smallest pressure was applied, the burnished samples looked similar to the sample after grinding, although the spatial parameters increased slightly (Figure 15 and Table 6). The surface roughness height decreased, and the biggest reduction of the Sq parameter (more than 50%) was acquired for the smallest burnishing width followed by the medium width. The Ssk parameter became more negative as a result of burnishing. Hybrid parameters, the Spc parameter, and peak density Spd also decreased.

For the burnishing pressure p of 15 MPa, only the surface burnished with the largest width looked similar to the surface after grinding (Figure 16). The view of the surface burnished with the smallest width changed more—the Str parameter increased to 0.502 due to burnishing; for the medium burnishing width, a change in the Str parameter was smaller. The Sal parameter increased due to burnishing. The increase in burnishing pressure from 5 to 15 MPa caused a reduction in the roughness height, and the smallest decrease corresponded to the largest burnishing width. The highest decrease in the Sq parameter was 69%. High decreases in hybrid parameters were caused by a decrease in the roughness height and an increase in the Sal parameter. The parameters Spc and Spd decreased as a result of ball burnishing compared to the initial surface after grinding. The Ssk skewness was still negative.

When the burnishing pressure p increased to 25 MPa, the surface height was slightly higher compared to that obtained at a pressure of 15 MPa (Figure 17 and Table 6). The surface texture was the most similar to the initial texture obtained after grinding when the burnishing width was the largest; in this case, the increases in the spatial parameters were the smallest. These parameters increased more for a smaller burnishing width. Hybrid parameters and the Spc parameter decreased due to burnishing, the highest reduction occurred for the smallest burnishing width, and the reduction in Spd reached 98%. The peak density increased for the largest burnishing width due to the large content of the high-frequency random component; for other burnishing widths, the peak density decreased due to burnishing.

For the highest burnishing pressure, the amount of the high-frequency random component was the highest when the burnishing width was the largest (Figure 18). The surface height was greater compared to the use of a burnishing pressure of 25 MPa; the highest amplitude was obtained for the smallest burnishing width. The skewness values were positive. As the burnishing spatial parameters increased, this growth was the smallest for the largest burnishing width. Due to the height decrease and increase in the Sal parameter, the parameters Sdq and Spc considerably decreased. The peak density decreased, and the changes were high for the smallest and medium burnishing widths.

The application of burnishing pressures of 5, 15, and 25 MPa led to similar roughness heights. Further pressure increases caused an increase in the amplitude of roughness. Spatial parameters increased due to ball burnishing. The lowest change in spatial characteristics occurred for the highest burnishing width.

**Figure 19:**
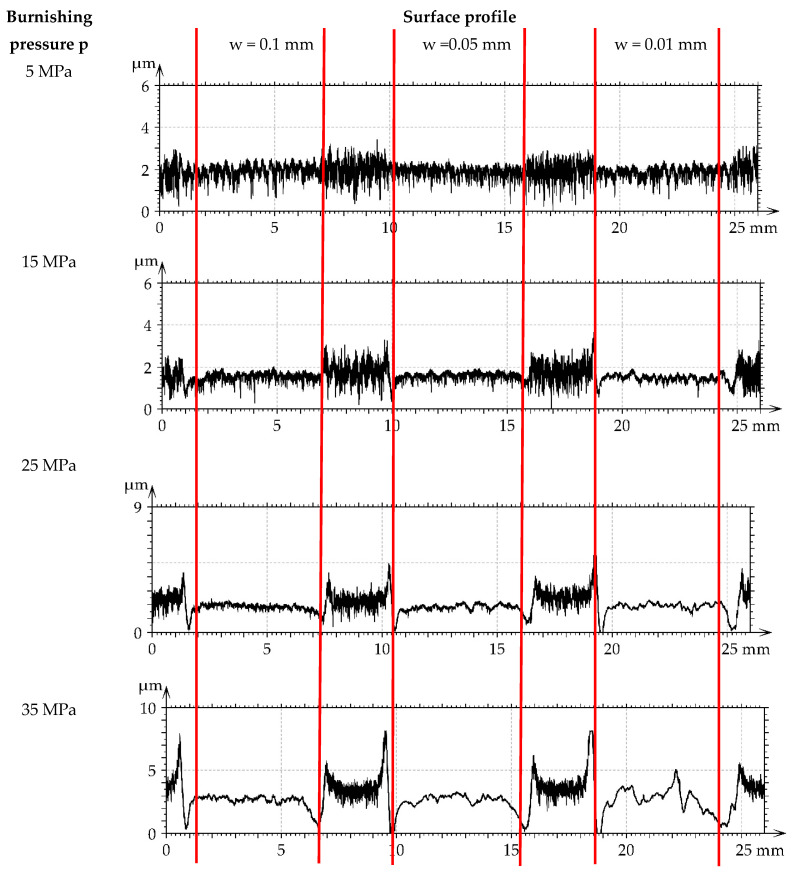
Roughness profiles performed through ground G1 and burnished samples.

Figure 19 presents the roughness profiles performed for the surfaces after grinding G1 and burnished samples. A reduction in surface height due to ball burnishing is visible. Due to burnishing, the roughness height decreased. This reduction depends on the initial surface texture before burnishing. It was the highest when the height of the machined texture was the biggest. The final average surface amplitudes of the initial surfaces M1 and G1 subjected to burnishing were similar, and the resulting smallest values of the Sq parameter were near 0.1 µm. However, burnishing of the M2 surface led to the minimum values of the Sq parameter near 0.22 µm. The smoother final amplitude of the burnished M1 surface compared to M2 may be caused by smaller values of the spatial parameters Str and Sal of the M2 surface compared to the M1 surface. The plastic deformation of the M1 surface was likely easier than that of the M2 surface. The possibility of obtaining a smoother surface due to burnishing is important because in [24,34,37], the lowest roughness height led to the smallest friction and wear.

When the M1 milled sample was burnished, the increase in the burnishing pressure led to a lower roughness height. Vaishya et al. [31] obtained similar findings. Different results were obtained for other burnished surfaces. In these cases, the smallest surface amplitude was achieved for the burnishing pressures of 15 and 25 MPa. The further increase in the burnishing pressure to 35 MPa caused growth in the height of the roughness. Perhaps too high burnishing pressure caused surface deterioration [38]. Swirad and Pawlus [25,34] obtained an increase in the height of the roughness when the burnishing pressure increased from 30 to 40 MPa. The application of burnishing widths of 0.01 and 0.05 mm caused similar results. However, for the largest burnishing width of 0.1 mm, the surface texture changes due to burnishing were slower compared to the smaller widths. This means that for the burnishing width of 0.1 mm, the spatial parameters were more similar to those of the initial surface compared to other surfaces (burnished at widths of 0.01 and 0.05 mm). This behavior was likely caused by the fact that this width was too large to overlap the neighboring paths. Furthermore, for the largest width when surfaces M2 and G1 were burnished, the resulting surface texture contained a large number of short-wavelength components. Therefore, the application of a burnishing width of 0.1 mm is not recommended.

During burnishing, not only height but also spatial characteristics changed. These changes typically increase with burnishing pressure growth. The final surface textures are characterized by the following ranges in spatial parameters: Sal 0.1–0.2 mm and Str 0.45–0.6. The burnished texture had a random or random-deterministic character, independent of the initial surface.

During burnishing, the hybrid parameters decreased. Their reductions were higher for a larger decrease in the amplitude and an increase in the correlation length Sal. The mean peak curvature Spc also decreased. Changes in peak density on the initial surface texture, spatial parameters, and presence of the high-frequency component were observed.

## 4. Conclusions

1. It was found that ball burnishing led to a decrease in surface height of up to 94%. These changes were larger for the higher amplitude of the surface texture prior to burnishing.
2. Due to ball burnishing hybrid parameters, Sdq, Sdr, and the mean peak curvature Spc decreased. The character of changes in the spatial parameters Sal and Str depended on the surface texture before burnishing. The increase in the burnishing pressure led to greater changes in spatial parameters.
3. The burnished surfaces without traces of the initial texture before burnishing had a random or random-deterministic character. They are characterized typically by Sal parameter values between 0.1 and 0.2 mm and Str parameter values between 0.45 and 0.6.
4. The effect of burnishing pressure on the height of the surface texture depended on the type of surface texture before burnishing. For a rough milled surface with a large correlation length, the increase in pressure from 5 to 35 MPa led to surface smoothing. For surfaces after milling and grinding of smaller roughness height and low correlation length, the burnishing pressures of 15 and 25 MPa caused the smallest amplitudes of surface textures. A further increase in burnishing pressure to 35 MPa led to an increase in roughness height.
5. The application of burnishing widths of 0.01 and 0.05 mm produced similar surface textures. A burnishing width of 0.1 mm is not recommended because it leads to a smaller change in the spatial character of surface texture during burnishing compared to smaller widths and may be related to the presence of high-frequency components.

## Figures and Tables

**Table 1:** Chemical composition of X37CrMoV5-1 steel.

Chemical Composition wt%							
C	Mn	Si	P	S	Cr	Mo	V
0.33–0.42	0.25–0.5	0.8–1.2	0–0.03	0–0.03	4.8–5.5	1.1–1.5	0.3–0.5

**Table 2:** Machining parameters of the milled samples.

	Spindle Speed	Feed	Inserts
Milled sample M1	300 rpm	200 mm/min	6
Milled sample M2	600 rpm	60 mm/min	1

**Table 3:** Burnishing parameters.

Parameter	Value
Burnishing strategy	Raster
Speed	400 mm/min
Ball	6 mm
Pressure p	5, 15, 25, 35 MPa
Burnishing width w	0.01, 0.05, 0.1 mm

**Table 4:** Surface texture parameters after milling (M1) and burnishing.

			Milled	Burnished											
				p = 5 MPa			p =15 MPa			p = 25 MPa			p = 35 MPa		
				Burnishing Width w, mm											
Height Parameters				0.01	0.05	0.1	0.01	0.05	0.1	0.01	0.05	0.1	0.01	0.05	0.1
Sq	µm		1.6	0.489	0.579	1.38	0.171	0.256	0.172	0.174	0.228	0.171	0.0887	0.091	0.133
		σ	0.024	0.040	0.047	0.146	0.014	0.023	0.022	0.028	0.037	0.027	0.013	0.015	0.027
Ssk			−0.18	−0.83	−0.75	−0.37	0.01	0.002	−0.21	−0.39	−0.07	−0.23	0.02	0.136	−0.02
		σ	0.004	0.068	0.061	0.040	0.002	0.001	0.028	0.064	0.012	0.036	0.006	0.022	0.007
Sku			2.42	3.36	3.67	2.46	2.43	2.5	3.14	3.56	2.89	3.3	3.57	2.97	3.05
		σ	0.040	0.274	0.300	0.261	0.198	0.225	0.410	0.436	0.472	0.512	0.641	0.485	0.747
Sp	µm		5.55	1.33	3.75	3.31	0.656	0.892	0.787	0.829	1.18	0.845	0.494	0.391	0.516
		σ	0.091	0.109	0.306	0.351	0.054	0.080	0.103	0.102	0.193	0.131	0.089	0.064	0.126
Sv	µm		4.77	1.97	5.36	4.41	0.621	0.984	0.859	1.05	1.42	1.02	0.384	0.326	0.599
		σ	0.078	0.161	0.438	0.468	0.051	0.088	0.112	0.129	0.232	0.158	0.069	0.053	0.147
Sz	µm		10.3	3.3	9.11	7.72	1.28	1.88	1.65	1.87	2.6	1.87	0.877	0.717	1.12
		σ	0.168	0.269	0.744	0.819	0.105	0.169	0.216	0.229	0.425	0.290	0.158	0.117	0.274
Sa	µm		1.31	0.384	0.455	1.12	0.142	0.21	0.137	0.136	0.183	0.135	0.0692	0.0728	0.106
		σ	0.021	0.031	0.037	0.119	0.017	0.019	0.018	0.017	0.030	0.021	0.012	0.012	0.026
Spatial parameters															
Sal	mm		0.107	0.08	0.069	0.116	0.142	0.142	0.122	0.099	0.136	0.019	0.141	0.159	0.116
		σ	0.002	0.007	0.006	0.012	0.017	0.013	0.016	0.012	0.022	0.003	0.025	0.026	0.028
Str			0.063	0.043	0.037	0.068	0.086	0.083	0.11	0.258	0.103	0.055	0.534	0.458	0.415
		σ	0.001	0.004	0.003	0.007	0.011	0.007	0.015	0.032	0.017	0.009	0.096	0.075	0.102
Hybrid parameters															
Sdq			0.072	0.044	0.049	0.058	0.013	0.021	0.022	0.034	0.039	0.040	0.007	0.009	0.012
		σ	0.001	0.007	0.004	0.006	0.002	0.002	0.003	0.004	0.006	0.006	0.001	0.002	0.003
Sdr	%		0.26	0.09	0.11	0.17	0.008	0.02	0.024	0.060	0.076	0.084	0.002	0.004	0.007
		σ	0.004	0.016	0.010	0.018	0.001	0.002	0.003	0.007	0.013	0.013	0.001	0.001	0.002
Feature parameters															
Spd	1/mm²		34.7	292	13.3	46.1	358	239	260	739	501	902	195	478	206
	σ		0.567	23.842	1.086	4.893	43.846	21.466	33.966	60.339	81.813	51.554	47.765	78.057	50.459
Spc	1/mm		59	43.6	50.6	56.2	7.35	18.7	14.3	29.5	38.1	32.6	3.63	4.39	5.82
	σ		0.963	7.120	4.131	5.965	0.900	1.680	1.868	3.613	6.222	3.993	0.889	0.717	1.426
Functional parameters															
Sk	µm		2.91	0.907	1.16	2.23	0.361	0.573	0.373	0.363	0.491	0.392	0.172	0.184	0.284
		σ	0.048	0.074	0.095	0.237	0.044	0.051	0.049	0.044	0.080	0.061	0.031	0.030	0.070
Spk	µm		0.83	0.145	0.173	0.478	0.087	0.127	0.109	0.126	0.156	0.117	0.0688	0.0663	0.102
		σ	0.014	0.012	0.014	0.051	0.011	0.011	0.014	0.015	0.025	0.018	0.012	0.011	0.025
Svk	µm		1.53	0.836	0.939	1.48	0.0884	0.144	0.174	0.235	0.18	0.192	0.0676	0.0592	0.103
		σ	0.025	0.068	0.077	0.157	0.011	0.013	0.023	0.029	0.029	0.030	0.012	0.010	0.025

**Table 5:** Surface texture parameters after milling (M2) and burnishing.

				Burnished											
Height Parameters			Milled	p = 5 MPa			p = 15 MPa			p = 25 MPa			p = 35 MPa		
				Burnishing Width w, mm											
				0.01	0.05	0.1	0.01	0.05	0.1	0.01	0.05	0.1	0.01	0.05	0.1
Sq	µm		1.070	0.250	0.273	0.398	0.293	0.269	0.225	0.407	0.227	0.236	0.665	0.465	0.376
		σ	0.026	0.027	0.025	0.042	0.024	0.033	0.029	0.066	0.037	0.048	0.098	0.076	0.075
Ssk			−0.489	−0.458	−0.471	−0.623	−0.271	−0.181	−0.372	−0.269	−0.190	−0.116	−0.106	−0.041	−0.106
		σ	0.004	0.068	0.061	0.040	0.002	0.001	0.028	0.064	0.012	0.036	0.006	0.022	0.007
Sku			2.100	3.840	3.460	2.950	3.170	3.570	3.830	3.420	3.180	2.960	2.890	3.370	2.690
		σ	0.040	0.274	0.300	0.261	0.198	0.225	0.410	0.436	0.472	0.512	0.641	0.485	0.747
Sp	µm		2.740	1.190	1.290	1.530	1.550	2.220	1.100	1.510	1.020	1.050	2.960	2.570	1.480
		σ	0.091	0.109	0.306	0.351	0.054	0.080	0.103	0.102	0.193	0.131	0.089	0.064	0.126
Sv	µm		4.290	2.190	1.670	2.250	1.710	2.620	2.020	2.100	2.100	1.330	3.290	2.950	2.200
		σ	0.078	0.161	0.438	0.468	0.051	0.088	0.112	0.129	0.232	0.158	0.069	0.053	0.147
Sz	µm		7.030	3.380	2.970	3.790	3.250	4.840	3.120	3.610	3.120	2.370	6.240	5.520	3.690
		σ	0.168	0.269	0.744	0.819	0.105	0.169	0.216	0.229	0.425	0.290	0.158	0.117	0.274
Sa	µm		0.886	0.194	0.216	0.324	0.233	0.213	0.177	0.321	0.180	0.190	0.536	0.365	0.305
		σ	0.021	0.031	0.037	0.119	0.017	0.019	0.018	0.017	0.030	0.021	0.012	0.012	0.026
Spatial parameters															
Sal	mm		0.02	0.02	0.02	0.03	0.12	0.14	0.03	0.18	0.13	0.05	0.16	0.18	0.07
		σ	0.002	0.007	0.006	0.012	0.017	0.013	0.016	0.012	0.022	0.003	0.025	0.026	0.028
Str			0.01	0.09	0.03	0.01	0.64	0.62	0.13	0.90	0.67	0.03	0.50	0.77	0.19
		σ	0.001	0.004	0.003	0.007	0.011	0.007	0.015	0.032	0.017	0.009	0.096	0.075	0.102
Hybrid parameters															
Sdq			0.12	0.06	0.07	0.08	0.05	0.05	0.06	0.02	0.03	0.04	0.02	0.02	0.03
		σ	0.001	0.007	0.004	0.006	0.002	0.002	0.003	0.004	0.006	0.006	0.001	0.002	0.003
Sdr	%		0.75	0.19	0.21	0.29	0.11	0.14	0.17	0.02	0.05	0.10	0.03	0.03	0.06
		σ	0.004	0.016	0.010	0.018	0.001	0.002	0.003	0.007	0.013	0.013	0.001	0.001	0.002
Feature parameters															
Spd	1/mm²		291	693	949	817	477	347	876	76	382	859	12	15	227
	σ		0.567	23.842	1.086	4.893	43.846	21.466	33.966	60.339	81.813	51.554	47.765	78.057	50.459
Spc	1/mm²		69	55	55	59	47	56	49	25	32	38	25	24	37
	σ		0.963	7.120	4.131	5.965	0.900	1.680	1.868	3.613	6.222	3.993	0.889	0.717	1.426
Functional parameters															
Sk	µm		2.16	0.56	0.64	0.87	0.67	0.60	0.54	0.80	0.48	0.60	1.08	0.81	0.93
		σ	0.048	0.074	0.095	0.237	0.044	0.051	0.049	0.044	0.080	0.061	0.031	0.030	0.070
Spk	µm		0.53	0.19	0.19	0.19	0.19	0.20	0.17	0.27	0.14	0.17	0.38	0.25	0.17
		σ	0.014	0.012	0.014	0.051	0.011	0.011	0.014	0.015	0.025	0.018	0.012	0.011	0.025
Svk	µm		1.57	0.35	0.34	0.52	0.30	0.27	0.28	0.39	0.22	0.23	0.47	0.30	0.29
		σ	0.025	0.068	0.077	0.157	0.011	0.013	0.023	0.029	0.029	0.030	0.012	0.010	0.025

**Table 6:** Surface texture parameters after grinding (G1) and burnishing.

				Burnished											
Height Parameters			Ground	p = 5 MPa			p = 15 MPa			p = 25 MPa			p = 35 MPa		
				Burnishing width w, mm											
				0.01	0.05	0.1	0.01	0.05	0.1	0.01	0.05	0.1	0.01	0.05	0.1
Sq	µm		0.417	0.201	0.223	0.281	0.131	0.127	0.167	0.190	0.136	0.148	0.266	0.174	0.175
		σ	0.007	0.021	0.020	0.030	0.011	0.016	0.022	0.031	0.022	0.030	0.039	0.028	0.035
Ssk			−0.338	−1.110	−0.897	−0.757	−0.057	−0.429	−0.491	0.031	0.004	−0.395	0.160	0.187	0.021
		σ	0.008	0.091	0.073	0.080	0.014	0.105	0.064	0.005	0.001	0.061	0.039	0.031	0.005
Sku			3.58	6.660	5.760	4.710	3.400	4.270	4.200	3.050	2.990	3.940	3.370	3.200	3.220
		σ	0.058	0.544	0.470	0.500	0.278	0.384	0.549	0.374	0.488	0.611	0.605	0.523	0.789
Sp	µm		1.77	0.933	1.020	1.160	0.765	0.763	0.804	0.811	0.713	1.010	1.480	0.861	1.320
		σ	0.029	0.076	0.083	0.123	0.062	0.069	0.105	0.099	0.116	0.157	0.266	0.141	0.323
Sv	µm		2.98	2.170	2.260	2.170	0.835	1.360	1.180	0.710	0.684	0.934	1.140	0.846	0.918
		σ	0.049	0.177	0.185	0.230	0.068	0.122	0.154	0.087	0.112	0.145	0.205	0.138	0.225
Sz	µm		4.75	3.100	3.280	3.330	1.600	2.130	1.980	1.520	1.400	1.940	2.630	1.710	2.240
		σ	0.078	0.253	0.268	0.353	0.131	0.191	0.259	0.186	0.229	0.301	0.472	0.279	0.549
Sa	µm		0.326	0.149	0.169	0.216	0.103	0.098	0.129	0.151	0.109	0.115	0.209	0.138	0.138
		σ	0.005	0.012	0.014	0.023	0.013	0.009	0.017	0.018	0.018	0.018	0.038	0.023	0.034
Spatial parameters															
Sal	mm		0.014	0.033	0.024	0.043	0.104	0.043	0.047	0.118	0.107	0.050	0.115	0.107	0.086
		σ	0.001	0.003	0.002	0.005	0.013	0.004	0.006	0.014	0.017	0.008	0.021	0.017	0.021
Str			0.0343	0.073	0.053	0.074	0.502	0.157	0.107	0.494	0.476	0.157	0.516	0.626	0.328
		σ	0.001	0.006	0.004	0.008	0.061	0.014	0.014	0.061	0.078	0.024	0.093	0.102	0.080
Hybrid parameters															
Sdq			0.095	0.041	0.048	0.054	0.023	0.029	0.035	0.013	0.018	0.034	0.013	0.013	0.026
		σ	0.002	0.007	0.004	0.006	0.003	0.003	0.005	0.002	0.003	0.005	0.002	0.002	0.006
Sdr	%		0.447	0.084	0.113	0.142	0.027	0.043	0.063	0.008	0.017	0.057	0.009	0.008	0.033
		σ	0.007	0.014	0.009	0.015	0.003	0.004	0.008	0.001	0.003	0.009	0.002	0.002	0.008
Feature parameters															
Spd	1/mm²		720	424	512	549	506	574	629	146	447	857	36	114	487
	σ		11.75	34.61	41.80	58.27	61.97	51.55	82.17	11.92	72.99	48.98	8.81	18.61	79.52
Spc	1/mm		64.5	38	42	45	21	27	30	11	16	29	12	12	26
	σ		1.053	6.140	3.413	4.723	2.547	2.398	3.971	1.347	2.613	3.515	2.890	1.894	6.271
Functional parameters															
Sk			1.01	0.394	0.501	0.656	0.307	0.284	0.366	0.399	0.325	0.335	0.572	0.396	0.397
	σ		0.016	0.032	0.041	0.070	0.038	0.026	0.048	0.049	0.053	0.052	0.103	0.065	0.097
Spk			0.341	0.130	0.155	0.177	0.114	0.108	0.130	0.155	0.111	0.118	0.238	0.143	0.153
	σ		0.006	0.011	0.013	0.019	0.014	0.010	0.017	0.019	0.018	0.018	0.043	0.023	0.037
Svk			0.533	0.340	0.339	0.427	0.140	0.167	0.224	0.160	0.116	0.198	0.221	0.124	0.161
	σ		0.009	0.028	0.028	0.045	0.017	0.015	0.029	0.020	0.019	0.031	0.040	0.020	0.039

## Data Availability

Data are contained within the article.
